# Supplementary material for: A systematic review of dengue controlled human infection studies: safety, viral kinetics and immunology
Source: PLoS Negl Trop Dis. 2026 Mar 12;20(3):e0014086. doi: 10.1371/journal.pntd.0014086 (PMC12998944; doi:10.1371/journal.pntd.0014086)
Supplement: S1 Text — (DOCX) [file pntd.0014086.s001.docx]

Search String Applied to MEDLINE, Embase and Cochrane

1. human challenge.mp.
2. controlled human infection.mp.
3. (“experimental” and “infection” and “human).mp.
4. (“wild-type virus” or “wildtype virus”).mp.
5. infect*.mp.
6. volunteer*.mp.
7. inoculat.mp.
8. 4 and 5
9. 4 and 7
10. 6 and 7
11. experimental*.mp
12. 7 and 11
13. 5 and 11
14. 1 or 2 or 3 or 8 or 9 or 10 or 12 or 13
15. Dengue.mp
16. 14 AND 15
