# Supplementary figures and images for: A systematic review of dengue controlled human infection studies: safety, viral kinetics and immunology
Source: PLoS Negl Trop Dis. 2026 Mar 12;20(3):e0014086. doi: 10.1371/journal.pntd.0014086 (PMC12998944; doi:10.1371/journal.pntd.0014086)

Risk of Bias Analysis Summary


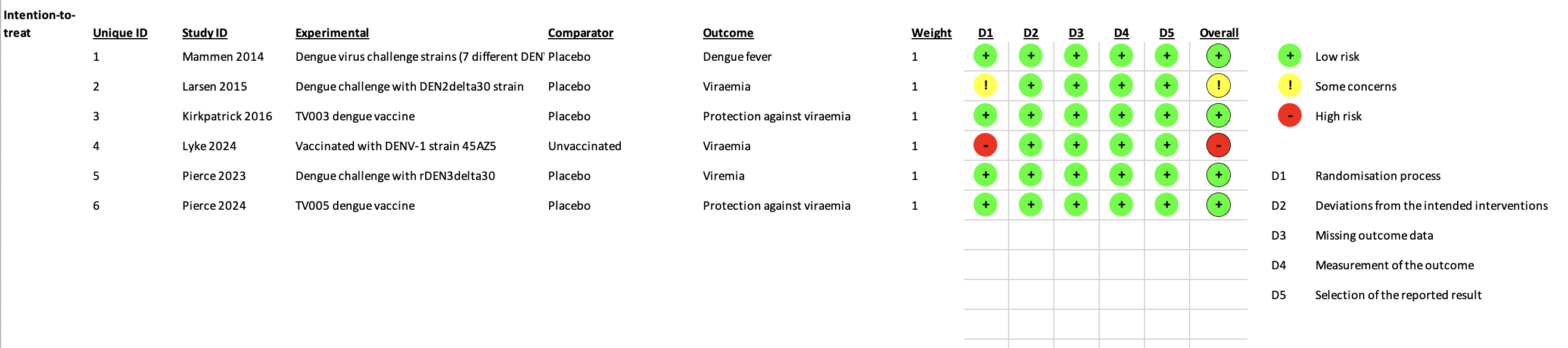

Supplement: S2 Text — (DOCX) [file pntd.0014086.s002.docx]
