# Supplementary material for: A systematic review of dengue controlled human infection studies: safety, viral kinetics and immunology
Source: PLoS Negl Trop Dis. 2026 Mar 12;20(3):e0014086. doi: 10.1371/journal.pntd.0014086 (PMC12998944; doi:10.1371/journal.pntd.0014086)
Supplement: S1 Table — (DOCX) [file pntd.0014086.s003.docx]

**Supplementary Table 1. Summary of study design and key clinical and biochemical findings amongst DCHIMs conducted in seronegative individuals**

|  | **Study, year** | **Location** | **Challenge agent; dose (n)** | **Attack rate:**  **1. Viraemia**  **2. *Signs/ symptoms** | **Symptoms** | **1. Peak viremia range (PFU/ml, mean)**  **2. Peak RNAemia range (GE/ml, mean)**  **3. Time to viremia/ RNAemia (TTV; mean)**  **4. Duration of viremia/RNAemia (mean)** | **Biochemical abnormalities** | **Follow up** | **SAE (Severe dengue)** |
| --- | --- | --- | --- | --- | --- | --- | --- | --- | --- |
| 1 | Durbin, The New England Journal of Medicine, 2025^Π^ | Johns Hopkins School of Public Health and the University of Vermont, USA | Δ30 rDEN3Δ30; 0.5ml 3 log 10 PFU/ml (8) | 1. 100%  2. 100% | Fever = 1 (12.5%)  Rash = 7 (87.5%)  Headache = 6 (75.0%)  Myalgia = 3 (37.5%) | 1. –  2. –  3. TTV: 2 – 5 days post challenge  4. – | NR | Outpatient study with some inpatient visits. Daily for the first 23 days, alternating between phone consult and physical visit between days 25-29, then visits on day 36, 43, 50, 57, 63, 70, 85 post infection | 0 (0) |
| 2 | Waikman, Nature Microbiology 2024 | Syracuse, New York, USA | SPA DENV-3 strain CH53489; 0.5ml 1.4 × 10^3^ PFU/ml (9) | 1. 100%  2. 100% | Fever = 8/9 (88.9%)  Rash = 7/9 (77.8%)  Headache = 8/9 (88.9%)  Myalgia 7/9 (77.8%) | 1. 3.72 – 7.48 log_10_  2. 4.50 – 8.85 log_10_  3. TTV: 3 – 6 (4.1) days post challenge  4. Duration: 4-11 (7.3) days | Elevated AST/ALT = 5/9 (55.6%)  Lymphopenia = 9/9 (100%)  Neutropenia = 7/9 (77.8%)  Thrombocytopenia = 4/9 (44.4%) | Outpatient study. Daily for the first 14 days, then every other day until 28 days post virus inoculation then days 90 and 180 post infection | 0 (0)  6 participants met protocol criteria for hospital admission |
| 3 | Pierce, medRxiv 2023 | Burlington, Vermont  and Baltimore, Maryland, USA | Δ30 rDEN3Δ30; 0.5ml 3 log PFU (10)  Placebo (4) | 1. DENV-3 = 100%; Placebo = 0%  2. DENV-3 = 80%; Placebo = 0% | DENV-3  Fever = 0/10 (0%)  Rash = 8/10 (80.0%)  Headache = 6/10 (60.0%)  Myalgia = 4/10 (40.0%)  Placebo  Fever = 0/4 (0%)  Rash = 0/4 (0%)  Headache = 3/4 (75.0%)  Myalgia = 1/4 (25.0%) | 1. 0.5 – 2.1 (1.8 ) log_10_  2. –  3. TTV: 3 – 6 (4.6) days post challenge  4. Duration: 3.4 days | DENV-3  Elevated AST/ALT = 0/10 (0%)  Neutropenia = 1/10 (10.0%)  Thrombocytopenia = 2/10 (20.0%)  Placebo  Elevated AST/ALT = 0/4 (0%)  Neutropenia = 0/4 (0%)  Thrombocytopenia = 0/4 (0%) | Outpatient study. Daily visits for 14 days and then on days 21, 28, 56, 90, and 180 | 0 (0) |
| 4 | Waickman, Science Translational Medicine 2022 | Syracuse, New York, USA | SPA DENV-1/45AZ5; 0.5ml 6.5 × 10^4^ PFU/ml (9) | 1. 100%  2. 100% | Fever = 5/9 (55.6%)  Rash = 5/9 (55.6%)  Headache = 8/9 (88.9%)  Myalgia = 7/9 (77.8%) | 1. 4.11 – 4.83 log_10_  2. 5.87 – 7.92 log_10_  3. TTV: 6 – 11 days post challenge  4. Duration: 8 days | Elevated AST/ALT = 3/9 (33.3%)  Leucopenia = 6/9 (66.7%)  Thrombocytopenia = 1/9 (11.1%) | Outpatient study. Daily visits for the first 14 days, then every other day until 28 days, and then on days 90 and 180 | 0 (0) |
| 5 | Endy, Journal of Infectious Diseases 2021 | Syracuse, New York, USA | SPA DENV-1/45AZ5; 0.5ml 3.25 × 10^3^ PFU/ml (6)  SPA DENV-1/45AZ5; 0.5ml 3.25 × 10^4^ PFU/ml (6) | 1. DENV-1 10^3^ = 83%; DENV-1 10^4^ = 100%  2. DENV-1 10^3^ = 83%; DENV-1 10^4^ = 100% | 3.25 × 10^3^ PFU/ml  Fever = 5/6 (83.3%)  Rash = 2/6 (33.3%)  Headache = 5/6 (83.3%)  Myalgia = 4/6 (66.7%)  3.25 × 10^4^ PFU/ml  Fever = 5/6 (83.3%)  Rash = 1/6 (16.6%)  Headache = 5/6 (83.3%)  Myalgia = 3/6 (50.0%) | 3.25 × 10^3^ PFU/ml  1. –  2. 7.2 log_10_  3. TTV: 5.4 days post challenge  4. Duration: 7.8 days  3.25 × 10^4^ PFU/ml  1. –  2. 6.70 log_10_  3. TTV: 6.3 days post challenge  4. Duration: 6.0 days | 3.25 × 10^3^ PFU/ml  Elevated AST/ALT = 2/6 (33.3%)  Leucopenia = 5/6 (83.3%)  Thrombocytopenia = 0/6 (0%)  3.25 × 10^4^ PFU/ml  Elevated AST/ALT = 1/6 (16.6%)  Leucopenia = 5/6 (83.3%)  Thrombocytopenia = 0/6 (0%) | Outpatient study. Daily visits for the first 14 days, then every other day until 28 days, and then on days 90 and 180 | 0 (0)  3 participants (2 low-dose, 1 high dose) met protocol criteria for hospital admission |
| 6 | Larsen, Vaccine 2015 | Baltimore, Maryland, USA | Δ30 rDEN2Δ30 Tonga strain; 10^3^ PFU (10)  Placebo (4) | 1. DENV-2 = 100%; Placebo = 0%  2. DENV-2 = 100%; Placebo = 0% | DENV-2  Fever = 0/10 (0%)  Rash = 8/10 (80.0%)  Headache = 6/10 (60.0%)  Myalgia = 2/10 (20.0%)  Placebo  Fever = 0/4 (0%)  Rash = 0/4 (0%)  Headache, Myalgia = NR | DENV-2  1. 1.5 – 3.3 (2.5) log_10_  2. –  3. TTV: 4.6 days post challenge  4. Duration: 5.8 days  Placebo  NA | DENV-2  Elevated AST/ALT = 0/10 (0%)  Neutropenia = 4/10 (40%)  Thrombocytopenia = 0/10 (0%)  Placebo  Elevated AST/ALT = NR  Neutropenia = 0/4 (0%)  Thrombocytopenia = 0/4 (0) | Outpatient study. Every other day visit for 16 days and then on days 21, 28, 56, and 180 | 0 (0) |
| 7 | Mammen, Vaccine 2014 | USA | SPA DENV-1 45AZ5 (2)  SPA DENV-2 S16803 (2)  SPA DENV-2 PR159 (1)  SPA DENV-3 CH53489 (3)  SPA DENV-4 341750 (3)  SPA DENV-4 H-241 (1)  Various PFU injected  Placebo (3) | 1. DENV-1 = 100%;  DENV-2 = 66.7%;  DENV-3 = 100%;  DENV-4 = 50.0%;  Placebo = 0%  2. DENV-1 = 100%;  DENV-2 = 0%;  DENV-3 = 100%;  DENV-4 = 0%;  Placebo = 0% | DENV-1  Fever = 2/2 (100%)  Rash = 2/2 (100%)  Headache = 2/2 (100%)  Myalgia = 2/2 (100%)  DENV-2  Fever = 0/3 (0%)  Rash = 2/3 (66.7%)  Headache = 3/3 (100%)  Myalgia = 2/3 (66.7%)  DENV-3  Fever = 3/3 (100%)  Rash = 3/3 (100%)  Headache = 3/3 (100%)  Myalgia = 3/3 (100%)  DENV-4  Fever = 2/4 (50.0%)  Rash = 1/4 (25.0%)  Headache = 4/4 (100%)  Myalgia = 3/4 (75.0%)  Placebo  Fever = 2/3 (67.7%)  Rash = 1/3 (33.3%)  Headache = 1/3 (33.3%)  Myalgia = 1/3 (33.3%) | DENV-1  1. NR  2. NR  3. TTV: 9-13 days post challenge  4. Duration: 12-14 days  DENV-2  1. NR  2. NR  3. TTV: 6-7 days post challenge  4. Duration: 12-13 days  DENV-3  1. NR  2. NR  3. TTV: 2-4 days post challenge  4. Duration: 8-9 days  DENV-4  1. NR  2. NR  3. TTV: 3-7 days post challenge  4. Duration: 10-12 days  Placebo  NA | DENV-1  Elevated AST/ALT = 0/2 (0%)  Leucopenia = 1/2 (50.0%)  Neutropenia = 1/2 (50.0%)  Thrombocytopenia = 0/2 (0%)  DENV-2  Elevated AST/ALT = 1/3 (33.3%)  Leucopenia = 2/3 (66.7%)  Neutropenia = 0/3 (0%)  Thrombocytopenia = 1/3 (33.3%)  DENV-3  Elevated AST/ALT = 2/3 (66.7%)  Leucopenia = 2/3 (66.7%)  Neutropenia = 1/3 (33.3%)  Thrombocytopenia = 3/3 (100%)  DENV-4  Elevated AST/ALT = 3/4 (75.0%)  Leucopenia = 2/4 (50.0%)  Neutropenia = 1/4 (25.0%)  Thrombocytopenia = 0/4 (0%)  Placebo  Elevated AST/ALT = 1/3 (33.3%) – attributed to alcohol consumption  Leucopenia = 0/3 (0%)  Neutropenia = 0/3 (0%)  Thrombocytopenia = 0/3 (0%) | Inpatient study. Admission from Days 1-14 post-challenge, then outpatient visits on days 20, 30, 40, 50 and 60 | 0 (0) |

NR = not reported, SPA = serial passage attenuated (WRAIR). All challenge agents were administered via subcutaneous injection. *Symptom attack rate was defined differently across studies; some reported a positive attack rate if participants had one or more symptom of dengue, whilst others only reported a positive attack rate if participants met a fixed pre-defined clinical syndrome suggestive of dengue infection. Some studies reported viremia using viral culture (PFU/ml), whilst some only reported RNAemia (GE/ml). Range of peak viral loads, incubation and duration of viremia are shown with means in brackets. Some studies only reported range without mean, or mean values without range and those are presented as such. Thrombocytopenia was defined as platelet count < 100 x 10^9^/L, neutropenia was defined as absolute neutrophil count < 1 x 10^9^/L. ^Π^ Only participants administered placebo followed by rDEN3Δ30 inoculation were included for analysis, whilst those administered Mosnodenvir were excluded.
