# Supplementary material for: A systematic review of dengue controlled human infection studies: safety, viral kinetics and immunology
Source: PLoS Negl Trop Dis. 2026 Mar 12;20(3):e0014086. doi: 10.1371/journal.pntd.0014086 (PMC12998944; doi:10.1371/journal.pntd.0014086)
Supplement: S2 Table — (DOCX) [file pntd.0014086.s004.docx]

**Supplementary Table 2. Summary of study design and key clinical and biochemical findings amongst DCHIMs conducted in previously vaccinated individuals**

|  | **Study, year** | **Location** | **Challenge agent; dose** | **Serostatus** | **Attack rate:**  **1. Viraemia**  **2. *Signs/ symptoms** | **Symptoms** | **1. Peak viremia range (PFU/ml, mean)**  **2. Peak RNAemia range (GE/ml, mean)**  **3. Time to viremia/ RNAemia (TTV; mean)**  **4. Duration of viremia/RNAemia (mean)** | **Biochemical abnormalities** | **Follow up** | **SAE (Severe dengue)** |
| --- | --- | --- | --- | --- | --- | --- | --- | --- | --- | --- |
| 1 | Lyke, Lancet ID 2024 | Baltimore, Maryland, USA | Tetravalent dengue purified vaccine + live-attenuated vaccine boost followed by SPA DENV-1 45AZ5; 0.5ml 6·5×10^3^ PFU/ml 27-65 months post booster (6)  Seronegative controls + SPA DENV-1 45AZ5; 0.5ml 6·5×10^3^ PFU/ml (4) | Seropositive (6)  Seronegative (4) | 1. Seropositive = 83.3%;  Controls = 100%  2. Seropositive = 83.3%;  Controls = 100% | Seropositive  Fever = 4/6 (66.6%)  Rash = 3/6 (50.0%)  Headache = 5/6 (83.3%)  Myalgia = 4/6 (66.6%)  Controls  Fever = 2/4 (50.0%)  Rash = 4/4 (100%)  Headache = 4/4 (100%)  Myalgia = 4/4 (100%) | Seropositive  1. -  2. 5.33 – 7.38 (7.32) log10  3. TTV: 4 – 6 (5.0) days post challenge  4. Duration: 7 – 10 (8.2) days  Controls  1. -  2. 6.78 – 7.68 (7.34) log10  3. TTV: 7 – 10 (8.0) days post challenge  4. Duration: 9 – 13 (10.5) days | Seropositive  Elevated AST/ALT = 4/6 (66.6%)  Leucopenia = 4/6 (66.6%)  Thrombocytopenia = 0/6 (0%)  Controls  Elevated AST/ALT = 3/4 (75.0%)  Leucopenia = 4/4 (100%)  Thrombocytopenia = 1/4 (25.0%) | Outpatient study.  Daily visits from 4-16 days post challenge until 2 consecutive negative RNA, then every 3 days until day 28, then day 90 and 180 | 3 fever ≥38·9°C , 1 headache, 1 transient grade 4 AST elevation (0) |
| 2a | Pierce, Journal of Clinical Investigation 2024 | Burlington, Vermont,  and Baltimore, Maryland, USA | TV005 followed by Δ30 rDEN2Δ30, Tonga strain; 10^3^ PFU 6 months later (21)  Placebo followed by Δ30 rDEN2Δ30, Tonga strain; 10^3^ PFU 6 months later (21) | Seropositive (21)  Seronegative (21) | 1. TV005 + DENV-2 = 0%; Placebo + DENV-2 = 100%  2. TV005 + DENV-2 = 0%; Placebo + DENV-2 = 100% | TV005 + DENV-2  Fever = 0/21 (0%)  Rash = 0/21 (0%)  Headache = 3/21 (14.3%)  Myalgia = 0/21 (0%)  Placebo + DENV-2  Fever = 0/21 (0%)  Rash = 21/21 (100%)  Headache = 8/21 (38.1%)  Myalgia = 6/21 (28.6%) | TV005 + DENV-2  NA  Placebo + DENV-2  1. 2.23 log_10_  2. -  3. TTV: 3 – 7 (5.0) days post challenge  4. Duration: 2 – 6 (4.6) days | TV005 + DENV-2  Elevated AST/ALT = 0/21 (0%)  Neutropenia = 0/21 (0%)  Thrombocytopenia = 0/21 (0%)  Placebo + DENV-2  Elevated AST/ALT = 2/21 (9.5%)  Neutropenia = 1/21(4.8%)  Thrombocytopenia = 0/21 (0%) | Outpatient study. Every other  day visit for 16 days and then on days 28, 56, 90, 180, and 360 | 0 (0) |
| 2b | Pierce, Journal of Clinical Investigation 2024 | Burlington, Vermont,  and Baltimore, Maryland, USA | TV005 followed by Δ30 rDEN3Δ30/Sleman 78; 10^4^ PFU 6 months later (23)  Placebo followed by Δ30 rDEN3Δ30/Sleman 78; 10^4^ PFU 6 months later (20) | Seropositive (23)  Seronegative (20) | 1. TV005 + DENV-3 = 0%; Placebo + DENV-3 = 85%  2. TV005 + DENV-3 = 9%; Placebo + DENV-3 = 100% | TV005 + DENV-3  Fever = 0/23 (0%)  Rash = 0/23 (0%)  Headache = 6/23 (26.1%)  Myalgia = 3/23 (13.0%)  Placebo + DENV-3  Fever = 1/20 (5.0%)  Rash = 20/20 (100%)  Headache = 11/20 (55.0%)  Myalgia = 7/20 (35%) | TV005 + DENV-3  NA  Placebo + DENV-3  1. 1.07 log_10_  2. –  3. TTV: 3 – 7 (4.6) days post challenge  4. Duration: 1 – 5 (2.0) days | TV005 + DENV-3  Elevated AST/ALT = 0/23 (0%)  Neutropenia = 2/23 (8.7%)  Thrombocytopenia = 0/23 (0%)  Placebo + DENV-3  Elevated AST/ALT = 0/20 (0%)  Neutropenia = 5/20 (25.0%)  Thrombocytopenia = 3/20 (15.0%) | Outpatient study. Every other  day visit for 16 days and then on days 28, 56, 90, 180, and 360 | 0 (0) |
| 3 | Kirkpatrick, Science Translational Medicine 2016 | Burlington, Vermont,  and Baltimore, Maryland, USA | TV003 followed by Δ30 rDEN2Δ30, Tonga strain; 10^3^ PFU 6 months later (21)  Placebo followed by Δ30 rDEN2Δ30, Tonga strain; 10^3^ PFU 6 months later (20) | Seropositive (21)  Seronegative (20) | 1. TV003 + DENV-2 = 0%; Placebo + DENV-2 = 100%  2. TV003 + DENV-2 = 0%; Placebo + DENV-2 = NR | TV003 + DENV-2  Fever = 0/21 (0%)  Rash = 0/21 (0%)  Headache = 5/21 (23.8%)  Myalgia = 1/21 (4.8%)  Placebo + DENV-2  Fever = 0/20 (0%)  Rash = 16/20 (80.0%)  Headache = 6/20 (30.0%)  Myalgia = 4/20 (20.0%) | TV003 + DENV-2  NA  Placebo + DENV-2  1. 2.3 log_10_  2. -  3. TTV: 4.7 days post challenge  4. Duration: 6.1 days | TV003 + DENV-2  Elevated AST/ALT = 0/21 (0%)  Neutropenia = 0/21 (0%)  Thrombocytopenia = 0/21 (0%)  Placebo + DENV-2  Elevated AST/ALT = 1/20 (5.0%)  Neutropenia = 4/20 (20.0%)  Thrombocytopenia = 2/20 (10.0%) | Outpatient study. Every other  day visit for 16 days and then on days 21, 28, 56, and 180 | 0 (0) |
| 4 | Sun, The Journal of Infectious Diseases 2013 | Washington, USA | Live attenuated tetravalent dengue vaccine (TDV) followed by SPA DENV-1 45AZ5; 0.5ml 10^3^ PFU 12-42 months later (5)  Seronegative controls given SPA DENV-1 45AZ5; 0.5ml 10^3^ PFU (2)  TDV followed by SPA DENV-3 CH53489 cl 24/28; 0.5ml 10^5^ PFU (5)  Seronegative controls given SPA DENV-3 CH53489 cl 24/28; 0.5ml 10^5^ PFU (2)  Revaccination in those who did not develop neutralising antibodies after TDV (2)◊ | Seropositive (10)  Seronegative (6) – 4 controls, 2 re-vaccinated | 1. TDV + DENV-1 = 20%  DENV-1 Controls = 50%;  TDV + DENV-3 = 60%  DENV-3 Controls = 100%  2. TDV + DENV-1 = 0%  DENV-1 Controls = 100%  TDV + DENV-3 = 60%  DENV-3 Controls = 100% | TDV + DENV-1  Fever = 1/5 (20.0%)  Rash = 0/5 (0%)  Headache = 4/5 (80.0%)  Myalgia = 1/5 (20.0%)  Controls + DENV-1  Fever = 2/2 (100%)  Rash = 1/2 (50.0%)  Headache = 2/2 (100%)  Myalgia = 1/2 (50.0%)  TDV + DENV-3  Fever = 3/5 (60.0%)  Rash = 2/5 (40.0%)  Headache = 3/5 (60.0%)  Myalgia = 3/5 (60.0%)  Controls + DENV-3  Fever = 2/2 (100%)  Rash = 2/2 (100%)  Headache = 2/2 (100%)  Myalgia = 2/2 (100%) | TDV + DENV-1  NA  Controls + DENV-1  1. –  2. 7.76 log_10_  3. TTV: 14 days post challenge  4. Duration: 3 days  TDV + DENV-3  1. –  2. 4.91 – 7.08 log_10_  3. TTV: 4 – 5 days post challenge  4. Duration: 3 – 7 days  Controls + DENV-3  1. –  2. 6.59 – 8.78 log_10_  3. TTV: 4 days post challenge  4. Duration: 7 – 8 days | TDV + DENV-1  Elevated AST/ALT = 1/5 (20.0%)  Neutropenia = 0/5 (0%)  Thrombocytopenia = 0/5 (0%)  Controls + DENV-1  Elevated AST/ALT = 1/2 (50.0%)  Neutropenia = 0/2 (0%)  Thrombocytopenia = 0/2 (0%)  TDV + DENV-3  Elevated AST/ALT = 4/5 (80.0%)  Neutropenia = 3/5 (60.0%)  Thrombocytopenia = 0/5 (0%)  Controls + DENV-3  Elevated AST/ALT = 2/2 (100%)  Neutropenia = 1/2 (50.0%)  Thrombocytopenia = 1/2 (50.0%) | Inpatient study. Admission from days 4-17 post-challenge, then outpatient visits on days 24, 30 and 60 | 0 (0)  2 participants challenged with DENV-3 developed severe LFT abnormalities which resolved within 30 days |

NR = not reported, SPA = serial passage attenuated (WRAIR). All seropositive individuals were seronegative prior to vaccination. ◊ Not included in our study analysis. *Symptom attack rate was defined differently across studies; some reported a positive attack rate if participants had one or more symptom of dengue, whilst others only reported a positive attack rate if participants met a fixed pre-defined clinical syndrome suggestive of dengue infection. Some studies reported viremia using viral culture (PFU/ml), whilst some only reported RNAemia (GE/ml). Range of peak viral loads, incubation and duration of viremia are shown with means in brackets. Some studies only reported range without mean, or mean values without range and those are presented as such. Thrombocytopenia was defined as platelet count < 100 x 10^9^/L, neutropenia was defined as absolute neutrophil count < 1 x 10^9^/L.
