# Supplementary material for: A systematic review of dengue controlled human infection studies: safety, viral kinetics and immunology
Source: PLoS Negl Trop Dis. 2026 Mar 12;20(3):e0014086. doi: 10.1371/journal.pntd.0014086 (PMC12998944; doi:10.1371/journal.pntd.0014086)
Supplement: Attachment — Submitted filename: PLOS Reviewer Report on Manuscript No.PNTD-D-25-01446.docx [file pntd.0014086.s007.docx]

PLOS Neglected Tropical Diseases

**Reviewer Submission**

**Reviewer Name:** Ibtisam Khalifa Hamed Al Maskari

**Date:** 15^th^ September 2025

**Manuscript Title:** *A systematic review of controlled human infection studies with attenuated dengue viruses: safety, viral kinetics and immunology*

**Authors:** Srishti Chhabra (Corresponding Author), Po Ying Chia, Yee-Sin Leo, and Barnaby Edward Young

**Cover Note**

Dear Dr. Puneet Bhatt, MD, PhD (Academic Editor), PLOS Neglected Tropical Diseases

Thank you for giving me the opportunity to review this research manuscript. I have examined the paper and provided my comments where necessary. My goal in this review was to improve the clarity, transparency, and overall quality of the work. My suggestions focused on the abstract, background, methods, statistical analysis, quality appraisal, tables, figures, and the presentation of abbreviations.

I believe this study addresses a highly relevant topic in the field of neglected tropical diseases. With the recommended revisions, it could make a significant contribution to the field.

I appreciate the chance to review this manuscript and hope my comments will help the authors strengthen their work. I wish them success in their revisions and in the eventual publication of this important study. I also want to sincerely thank Dr. Puneet Bhatt, MD, PhD, Academic Editor at PLOS Neglected Tropical Diseases, for allowing me to serve as a reviewer and contribute to the assessment of this valuable submission.

Regards,
Ms. Ibtisam Khalifa Hamed Al-Maskari

**Email:** [s143505@student.squ.edu.om](mailto:s143505@student.squ.edu.om)

**Personal email:** ibtisamalmaskari2020@gmail.com

**Reviewer Report**

**Manuscript Title:** *A systematic review of controlled human infection studies with attenuated dengue viruses: safety, viral kinetics and immunology*

**Dear Authors:** Srishti Chhabra **(Corresponding Author),** Po Ying Chia, Yee-Sin Leo, and Barnaby Edward Young:

I would like to sincerely thank you for your efforts and the considerable work you have invested in preparing this manuscript. The topic you have addressed, knowledge, and practices towards the use of Dengue controlled human infection models (DCHIM) in practice, is both timely and important. Your study contributes to a rapidly evolving area in healthcare, and with further refinement.

The PRISMA 2020 expanded checklist was used as guidance for evaluating this work:

1. **Title:**

Although it starts with the words' Systematic Review,' it mainly focuses on the dengue-controlled human infection challenge models (DCHIM), *which do not appear in the title*. My suggestion is to include DCHIM, since it covers safety, viral kinetics, and immunology, from the start to maintain the focus of the review and to ensure that this report has an informative title that provides key information about the main objective or question the review addresses (e.g., the population(s) and intervention(s) the review addresses.

**Affiliation (Institutional affiliation):** this part needs to be included in this review.

**The ORCID ID for the first author:** Recommended for scientific work to have ORCID ID.

**Abstract:**

**PRISMA 2020 for Abstracts Checklist was used in this review.**

a. Background: The authors clearly stated that this review was done to review the clinical, biochemical, and immunologic features of DCHIM in background suction**.**
b. Method: Clearly, they stated that they developed & registered a protocol for this review (register details written). Inclusion criteria are clear. The date when each was last searched is also included (January 2000 - April 2024).
c. *Specify the methods used to assess risk of bias in the included studies.*
d. *Specify the methods used to present and synthesize results.*
e. **Result: 10 published studies were included, with a total of 225 participants aged 18-50 years i**ncluded.
f. ***No Discussion section found in the abstract (Accepted according to the journal guideline).***
2. **Introduction:**
a. Rational: It started with the current state of knowledge and its uncertainties. Clearly stated the main purpose of this study, and as it’s the first study in its scope, it's not an update or replication of a particular systematic review.
b. Objective: The aim of this study was clearly mentioned.
3. **Methods:**
a. Eligibility Criteria: Thank you for stating that this review was conducted in accordance with PRISMA guidelines. The study characteristics used to determine whether a study was eligible for inclusion in the review followed the PICOST framework. Additionally, clarity on eligibility criteria related to report characteristics, such as year of publication, language, and report status, was met.
b. In ***Databases, Search Construct,*** *were all of these k*ey terms done in EMBASE and then also used in MEDLINE databases?

Suggestion:

| **Database** | **Key Terms** |
| --- | --- |
| EMBASE |  |
| MEDLINE |  |

c. ‘‘Disagreements were resolved by consensus or appeal to a third senior reviewer (P.Y.C.). It would be better to give an example and explain what disagreements they faced and resolved. For the work transparency.
d. What risks of bias assessment tools, effect measures, and any sensitivity analyses were utilized in this review?
4. **Results:**
a. **Table 1. Summary of study characteristics and attack rate:** (Table 1. Summary of the study characteristics and attack rate are very clear and well explained in this table. However, under intervention, many new abbreviations related to DENV strains are noted that are not explained beforehand and may confuse the reader. I recommend inserting the full abbreviations or providing some explanation as a footnote to effectively communicate the findings and keep it self-explanatory.
5. **Discussion:**

Clearly discussed implications of the results for practice and policy. Also, recommendations for future research were included.

6. **Limitation:**
1. *Thank you for this excellent work. I would like you to add a clear section for* ***Conclusion/Summary of findings****, with limitations, and implications detailed in your review, to highlight the key points and their implications.*
2. *Thank you for stating that this review protocol was developed and registered with PROSPERO [CRD42024558534. According to PLOS Neglected Tropical Diseases and PRISMA guidelines, it's highly recommended to provide registration information for the review, including register name and registration number, or state that the review was not registered to ensure transparency and reduce bias. I would recommend adding* ***a link to the protocol*** *for easy access.*
3. ***Abbreviations:*** *There is no abbreviation section noted in this review. It is highly recommended to include a table of abbreviations as guidance for the reader. These terms could be included in the abbreviation table: (DCHIMs, DENV, PROSPERO, AST, ALT, WRAIR, NIH, Δ30 strains, SPA).*

I appreciate the opportunity to review your work and hope my comments will help you strengthen the manuscript. I wish you success with your revisions and the eventual publication of this important study.

**Overall Recommendation**

This manuscript addresses an important topic related to Dengue fever since it is an ongoing problem. With the suggested improvements, it could make a significant contribution to the field. The revisions will improve clarity, transparency, and the overall rigor of the study. This document requires another evaluation by the second reviewer for a thorough assessment.
