# Supplementary material for: A systematic review of dengue controlled human infection studies: safety, viral kinetics and immunology
Source: PLoS Negl Trop Dis. 2026 Mar 12;20(3):e0014086. doi: 10.1371/journal.pntd.0014086 (PMC12998944; doi:10.1371/journal.pntd.0014086)
Supplement: Attachment — Submitted filename: Response to Reviewers.docx [file pntd.0014086.s010.docx]

**Response to Reviewers**

**Reviewers' comments:**

Reviewer's Responses to Questions

**Key Review Criteria Required for Acceptance?**

As you describe the new analyses required for acceptance, please consider the following:


**Methods**

-Are the objectives of the study clearly articulated with a clear testable hypothesis stated?

-Is the study design appropriate to address the stated objectives?

-Is the population clearly described and appropriate for the hypothesis being tested?

-Is the sample size sufficient to ensure adequate power to address the hypothesis being tested?

-Were correct statistical analysis used to support conclusions?

-Are there concerns about ethical or regulatory requirements being met?

Reviewer #1: In this review, its clearly stated the main purpose of this study, and as it’s the first study in its scope, it's not an update or replication of a particular systematic review. The population described and appropriate to the hypothesis being tested.

Reviewer #2: (No Response)

Reviewer #3: 1. This was assessed for rigor, and according to the authors, it followed the guidelines of PRISMA and was also registered with PROSPERO. checked for bias

2. Search terms were appropriate for identifying controlled human studies, but it didn't include “attenuated dengue virus.”

The search term “attenuated dengue virus” was not used as we were looking for challenge studies, instead of attenuated strains more commonly used for attenuated dengue vaccines

3. Why was the search of the authors limited to just EMBASE and MEDLINE? Why didn’t they consider Cochrane, Google Scholar, etc., for a more comprehensive result?

We have updated the search to include Cochrane and have also re-run the search until December 2025 to include any new studies. Reference tracking was performed for a more comprehensive result.

4. For the quality assessment and for risk of bias for controlled trials, did the authors use the Cochrane Risk of Bias Tool (RoB 2.0)? This wasn’t stated in the methodology.

Thank you for the comment, and we agree this is an important assessment to include in a systematic review. We have added this information to our methodology under “Databases, Search Construct and Study Selection” that Cochrane Risk of Bias Tool (RoB 2.0) was used to assess risk of bias in all included randomised controlled trials. Results from Risk of Bias Assessment are included in the Supplementary data.

5. Take care to note the ethical oversight/protocol of the original studies.

All studies were approved by their relevant ethics boards and informed consent was obtained from all recruited participants

6. Route and dose were not clearly stated in the methods, or did I miss it?

The doses of the dengue challenge strains are stated in Table 1. We have added that all dengue challenge strains were administered via subcutaneous injection in the Table 1 legend.

**Results**


-Does the analysis presented match the analysis plan?

-Are the results clearly and completely presented?

-Are the figures (Tables, Images) of sufficient quality for clarity?

Reviewer #1: Yes, overall results section is clear, and completely presented. Table 1. Summary of study characteristics and attack rate: (Table 1. Summary of the study characteristics and attack rate are very clear and well explained in this table. However, under intervention, many new abbreviations related to DENV strains are noted that are not explained beforehand and may confuse the reader. I recommend inserting the full abbreviations or providing some explanation as a footnote to effectively communicate the findings and keep it self-explanatory.

We have added the nomenclature of the dengue virus strains in the table description.

Reviewer #2: (No Response)

Reviewer #3: Yes, the analysis presented matches the plan.
Results are well presented
The tables and figures have clarity

1. Why was the SPA DENV-1 seropositive value for the thrombocytopenia lower than the seronegative? Kindly include it in the discussion.

Although the percentage of participants who developed thrombocytopenia was higher in seropositive participants than seronegative, the number of participants included are small, and this did not meet statistical significance to make definite conclusions about this trend.

2. Line 242 Endy et al describe one subject who developed high neutralizing antibody titers at study days 90 and 180 despite having undetectable viremia by polymerase chain reaction (PCR). Can you discuss this further? It would be reasonable.

This observation is interesting, and we suspect may have been due to low levels of systemic viral replication below the limit of detection. The reasons for this are not known though or described in the study so any further discussion would be speculative

3. Line 259 kindly discusses the reason for lower doses of SPA causing higher or longer viremia than the higher.

The reasons for this are not known and is likely a complex interaction of viral kinetics and host immunology which has not been elucidated. We have included this in the discussion.

Line 268: the association between post-inoculation viremia levels and symptoms was not observed consistently. Why? Kindly add to the discussion.

The reasons for this are not known and is likely a complex interaction of viral kinetics and host immunology which has not been elucidated. We have included this in the discussion.

4. Line 278 DENV-1 model described by Lyke et al. demonstrated earlier viremia (day 5 vs day 8; p=0.007) with a higher peak viral load and area under the curve in the vaccinee group compared to seronegative controls: implications of this come in the discussions

Based on the above observation of risk of antibody dependent enhancement with SPA strains, we have stated in the discussion that Δ30 strains may be more suitable as models of secondary dengue in endemic regions rather than SPA strains which were used by Lyke et al.

5.283 authors were already discussing the results; I feel this should have come as part of the discussion, comparing two studies in a discussion style.

In the results, we describe the results of the 2 studies which showed contrasting results. The implications of this are further described in the discussion section.

**Conclusions**


-Are the conclusions supported by the data presented?

-Are the limitations of analysis clearly described?

-Do the authors discuss how these data can be helpful to advance our understanding of the topic under study?

-Is public health relevance addressed?

Reviewer #1: the review finished with a comprehensive discussion section that is clearly discussed implications of the results for practice and policy. Also, recommendations for future research were included. However, there is no Conclusion/ summary section in this review.

Thank you for this excellent work. I would like you to add a clear section for Conclusion/Summary of findings, with limitations, and implications detailed in your review, to highlight the key points and their implications.

We have added a conclusion which states the implications of the review, which are

(a) DCHIMs are safe and consistently result in viremia
(b) They can be used to study the efficacy of anti-virals and vaccinations
(c) NIH strains may be preferred models in the context of secondary dengue

Reviewer #2: (No Response)

Reviewer #3: 1. Yes, the conclusions are supported by data provided.

2. Limitations were well defined

3. The authors made a fair attempt at the public health relevance but need to discuss more with clarity how their review can benefit and advance our understanding of the topic and also explain the public health impact of this work.

We have added a conclusion which states the implications of the review, which are

(a) DCHIMs are safe and consistently result in viremia
(b) They can be used to study the efficacy of anti-virals and vaccinations
(c) NIH strains may be preferred models in the context of secondary dengue

**Reviewer Report**

**Manuscript Title:** *A systematic review of controlled human infection studies with attenuated dengue viruses: safety, viral kinetics and immunology*

**Dear Authors:** Srishti Chhabra **(Corresponding Author),** Po Ying Chia, Yee-Sin Leo, and Barnaby Edward Young:

I would like to sincerely thank you for your efforts and the considerable work you have invested in preparing this manuscript. The topic you have addressed, knowledge, and practices towards the use of Dengue controlled human infection models (DCHIM) in practice, is both timely and important. Your study contributes to a rapidly evolving area in healthcare, and with further refinement.

The PRISMA 2020 expanded checklist was used as guidance for evaluating this work:

1. Although it starts with the words' Systematic Review,' it mainly focuses on the dengue-controlled human infection challenge models (DCHIM), *which do not appear in the title*. My suggestion is to include DCHIM, since it covers safety, viral kinetics, and immunology, from the start to maintain the focus of the review and to ensure that this report has an informative title that provides key information about the main objective or question the review addresses (e.g., the population(s) and intervention(s) the review addresses.

We have changed the title to “A systematic review of dengue controlled human infection studies: safety, viral kinetics and immunology”

2. **Affiliation (Institutional affiliation):** this part needs to be included in this review.

Affiliations are included in the title page and submission portal

3. **The ORCID ID for the first author:** Recommended for scientific work to have ORCID ID.

The ORCID ID for the first author is included in the submission portal

4. In ***Databases, Search Construct,*** *were all of these k*ey terms done in EMBASE and then also used in MEDLINE databases?

Yes, all the terms stated were included in Embase, MEDLINE and now Cochrane data bases

Suggestion:

| **Database** | **Key Terms** |
| --- | --- |
| EMBASE |  |
| MEDLINE |  |

5. ‘‘Disagreements were resolved by consensus or appeal to a third senior reviewer (P.Y.C.). It would be better to give an example and explain what disagreements they faced and resolved. For the work transparency.

There were no disagreements that required appeal to a third senior reviewer, however this provision was in place in case required.

6. What risks of bias assessment tools, effect measures, and any sensitivity analyses were utilized in this review?

The Cochrane Risk of Bias Tool was used for all randomised controlled trials included in this study. Effect measures and sensitivity analysis were not carried out as the numbers studied in each dengue strain are too small.

7. **Results: Table 1. Summary of study characteristics and attack rate:** (Table 1. Summary of the study characteristics and attack rate are very clear and well explained in this table. However, under intervention, many new abbreviations related to DENV strains are noted that are not explained beforehand and may confuse the reader. I recommend inserting the full abbreviations or providing some explanation as a footnote to effectively communicate the findings and keep it self-explanatory.

We have added the nomenclature of the dengue virus strains and vaccines in the table description.

8. *Thank you for this excellent work. I would like you to add a clear section for* ***Conclusion/Summary of findings****, with limitations, and implications detailed in your review, to highlight the key points and their implications.*

We have added a conclusion which states the implications of the review, which are

(a) DCHIMs are safe and consistently result in viremia
(b) They can be used to study the efficacy of anti-virals and vaccinations
(c) NIH strains may be preferred models in the context of secondary dengue
9. *Thank you for stating that this review protocol was developed and registered with PROSPERO [CRD42024558534. According to PLOS Neglected Tropical Diseases and PRISMA guidelines, it's highly recommended to provide registration information for the review, including register name and registration number, or state that the review was not registered to ensure transparency and reduce bias. I would recommend adding* ***a link to the protocol*** *for easy access.*

The PROSPERO registration number, CRD42024558534, has been included

10. ***Abbreviations:*** *There is no abbreviation section noted in this review. It is highly recommended to include a table of abbreviations as guidance for the reader. These terms could be included in the abbreviation table: (DCHIMs, DENV, PROSPERO, AST, ALT, WRAIR, NIH, Δ30 strains, SPA).*

A table of abbreviations has been included in the supplementary data

I appreciate the opportunity to review your work and hope my comments will help you strengthen the manuscript. I wish you success with your revisions and the eventual publication of this important study.

**Overall Recommendation**

This manuscript addresses an important topic related to Dengue fever since it is an ongoing problem. With the suggested improvements, it could make a significant contribution to the field. The revisions will improve clarity, transparency, and the overall rigor of the study. This document requires another evaluation by the second reviewer for a thorough assessment.

**Reviewer Name:** Ibtisam Khalifa Hamed Al Maskari

**Date:** 15^th^ September 2025
